# A Case of Severe Refractory Immune Thrombocytopenia Exacerbated by Respiratory Syncytial Virus Infection

**DOI:** 10.7759/cureus.93841

**Published:** 2025-10-04

**Authors:** James Kim, Vicken Khazar, Rebecca E McIver, Benjamin Ascherman, Maria J Nieto

**Affiliations:** 1 Hematology and Oncology, Donald and Barbara Zucker School of Medicine at Hofstra/Northwell, Hempstead, USA; 2 Medicine, Rutgers Robert Wood Johnson Medical School, New Brunswick, USA

**Keywords:** cyclosporine, ecchymosis, immune thrombocytopenia, microcytic anemia, respiratory syncytial virus

## Abstract

Immune thrombocytopenia (ITP) is an immunological phenomenon in which antibodies target platelet surface proteins, resulting in platelet destruction, as well as platelet opsonization and clearance by splenic and hepatic macrophages. Antibodies and cytotoxic T cells may also directly target megakaryocytes in the bone marrow, thereby compromising platelet production and further lowering platelet counts. This disease is largely idiopathic but may also result secondary to autoimmune disorders, malignancies, medication side effects, and recent infections. Currently, the understanding of infections and thrombocytopenia is poorly understood. There are no current well-described reports of severe ITP exacerbations due to respiratory syncytial virus (RSV) infection, especially in a relatively healthy adult with no significant hematologic or oncologic history. This case report describes a 57-year-old woman with no significant past medical history who presented to the ED with a three-day duration of epistaxis in the setting of upper respiratory tract infection symptoms, as well as easy bruising over the past several months. Initial bloodwork in the ED revealed severe thrombocytopenia and anemia. Treatment was initiated, starting with platelet and packed red blood cell infusion. Her anemia resolved, but her thrombocytopenia persisted and remained largely refractory to multiple first-line treatments, including intravenous immunoglobulin therapy, steroids, and thrombopoietin receptor agonists. It was only after a four-day treatment with cyclosporine that her platelet count was normalized. This case report describes an unusual presentation of severe, refractory ITP that may have been exacerbated by an RSV infection.

## Introduction

Immune thrombocytopenia (ITP) has an estimated global prevalence of 10 per 100,000 persons in the USA and mostly affects adults aged >55 years [1]. It is an immunological phenomenon in which antibodies target platelet surface proteins, such as GP IIb/IIIa, Ib/IIa, and VI [2]. The formation of antibodies remains somewhat unclear, although current literature suggests molecular mimicry, somatic mutations, or defects in autoreactive B cell elimination as probable causes [3]. It is also sometimes associated with viral infections such as HIV, hepatitis C, cytomegalovirus, and varicella-zoster virus. Antibody binding to platelet surface proteins results in platelet sequestration within the spleen and destruction by splenic macrophages, as well as peripheral destruction via complement [3]. This leads to thrombocytopenia and may result clinically in recurrent, easily forming ecchymoses and petechiae. Other findings include mucosal bleeding such as gingival bleeds, epistaxis, menorrhagia, and occult gastrointestinal bleeding. Feared, severe complications include intracranial hemorrhage or severe gastrointestinal bleeds [4]. Other complications include frequent infections, such as candidiasis and various gram-positive and gram-negative bacteria, as ITP is primarily managed with immunosuppressive therapies, including corticosteroids, intravenous immunoglobulin (IVIG), rituximab, thrombopoietin receptor agonists (TPO-RAs), and, in severe cases, splenectomy [5]. This case report represents a unique case of a respiratory syncytial virus (RSV) infection associated with acute ITP. Furthermore, the patient interestingly remained refractory to many of the first-line management options for ITP, such as corticosteroids, IVIG, and rituximab.

## Case presentation

**Figure 1:**
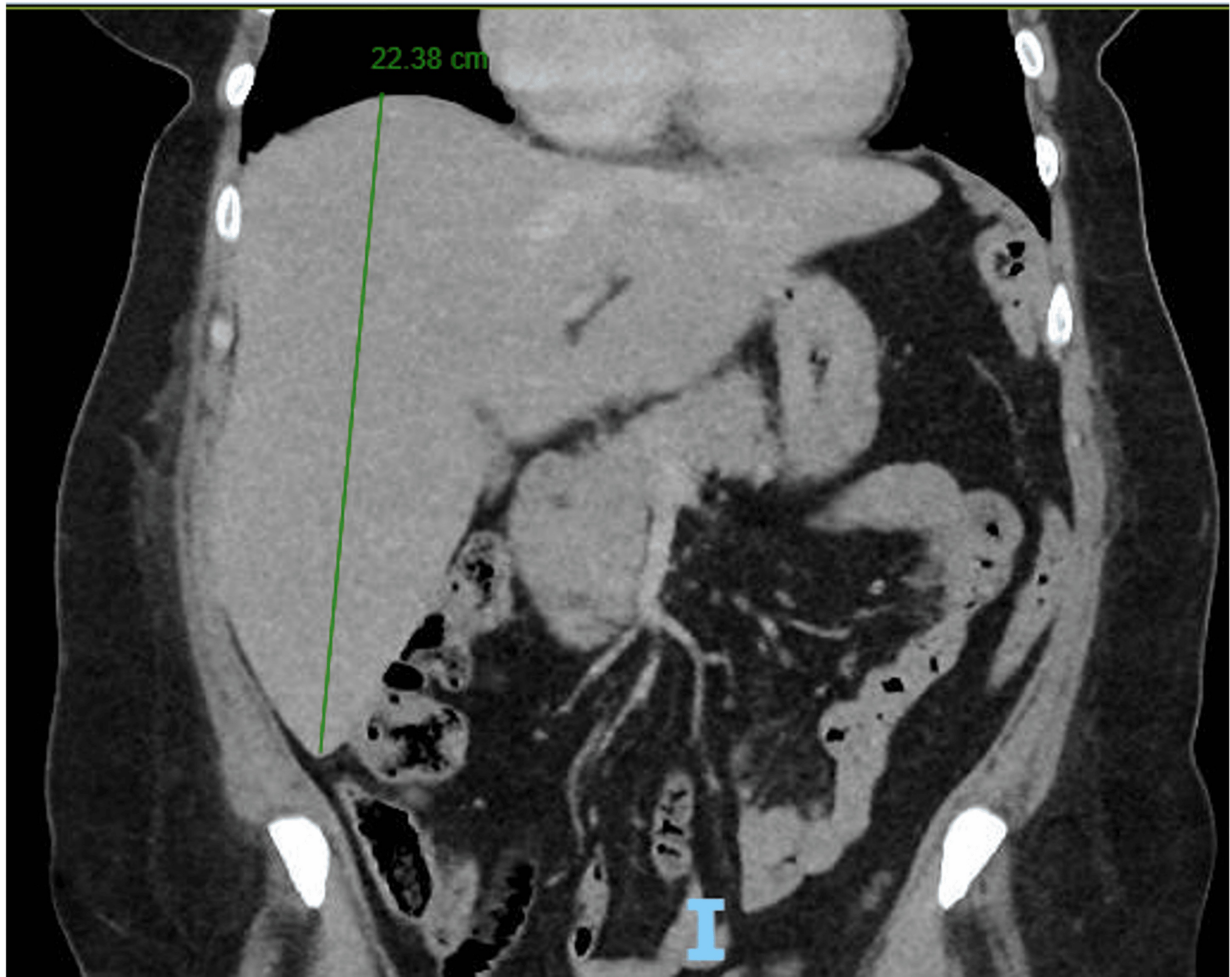
CT scan of the chest, abdomen, and pelvis for hepatomegaly Coronal view of the chest, abdomen, and pelvis. Liver measuring 22.38 cm (green line) I, Inferior

**Figure 2:**
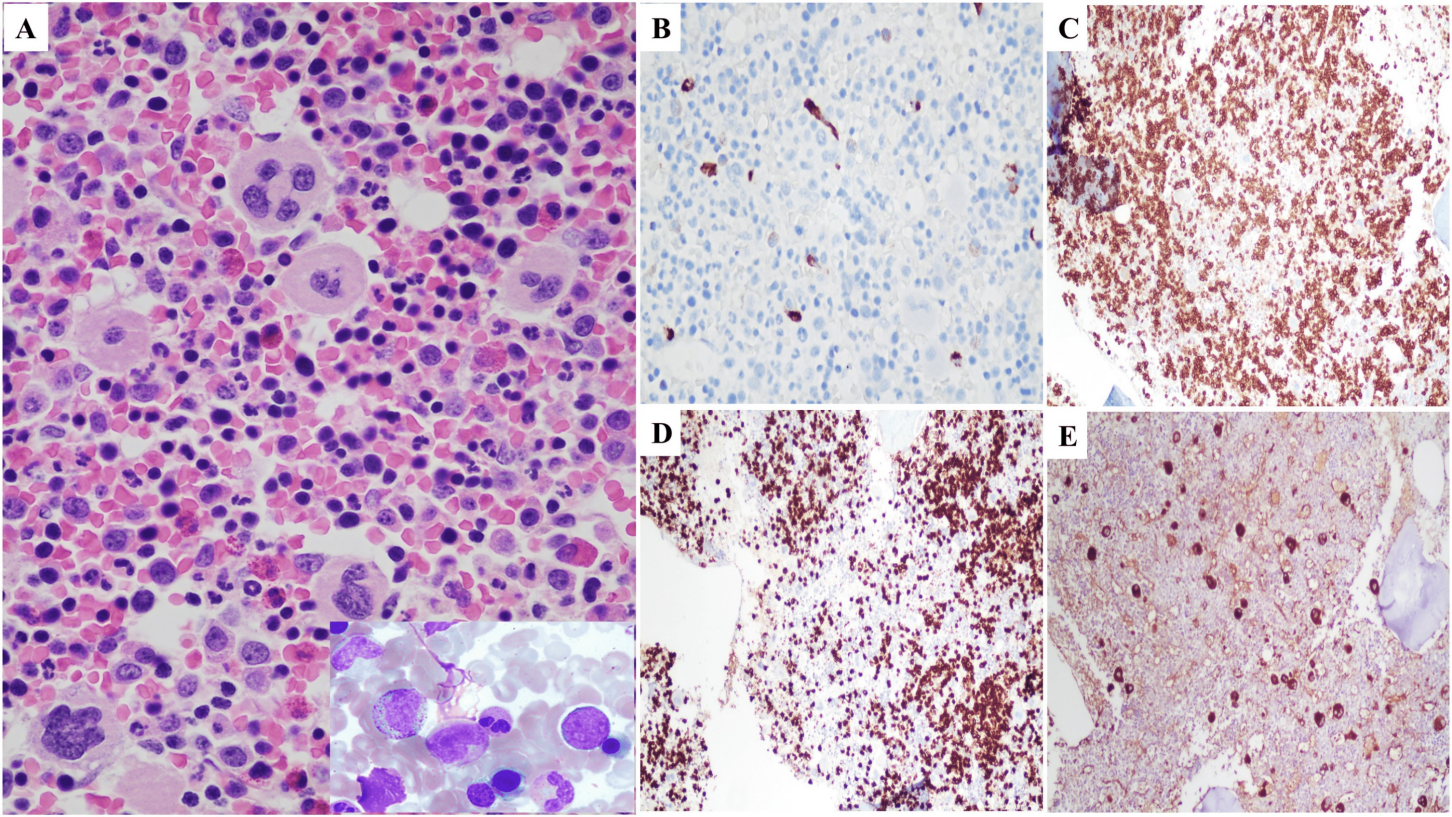
Composite photomicrograph of bone marrow biopsy and aspirate A. Bone marrow biopsy with inset of bone marrow aspirate smear (showing hypercellular marrow with erythroid predominant trilineage hematopoiesis, maturation, and mild megakaryocytosis) B. CD34 stain C. CD71 stain for erythroid cells D. Myeloperoxidase stain for erythroid cells E. Factor VIII stain for megakaryocytes

A 57-year-old woman with no known past medical history presented to the ED with epistaxis of three days' duration, intermittent dyspnea, and a mild cough. She reported having episodes of epistaxis once a month for the past year, but these episodes were mild and self-resolving. She also reported easy bruising for the last few months. Of note, she had not sought medical care for several years. Her vital signs included a temperature of 99.3°F, a heart rate of 100 beats per minute, blood pressure of 127/67 mmHg, a respiratory rate of 16 breaths per minute, and an oxygen saturation level of 97% on room air. On physical examination, she had dried blood present in her left naris. Her initial lab work (Table 1) was remarkable for leukocytosis (WBC count: 20.01 K/μL with 82.1% neutrophils), severe microcytic anemia (hemoglobin: 2.5 g/dL, hematocrit: 10.9%, and mean cell volume: 67.3 fL), thrombocytopenia (platelets: 7 K/μL), elevated uric acid (8.2 mg/dL), elevated lactate dehydrogenase (566 U/L), and transaminitis (aspartate aminotransferase: 252 U/L and alanine aminotransferase: 222 U/L). CT scan of the chest, abdomen, and pelvis was remarkable for hepatomegaly (22.38 cm) (Figure 1). Iron studies demonstrated low iron levels (24 µg/dL), a normal total iron-binding capacity (334 µg/dL), a low percent saturation (7%), and a normal ferritin level (22 ng/mL). Her severely low hemoglobin and hematocrit levels prompted further workup to rule out hemolysis; her haptoglobin level was 86 mg/dL. To rule out paroxysmal nocturnal hemoglobinuria (PNH), phosphatidylinositol-linked antigen testing was performed, demonstrating normal immunophenotyping results with no PNH clone detected in RBCs, granulocytes, or monocytes. The peripheral smear showed severely microcytic and hypochromic RBCs, no schistocytes, a few atypical monocytes with no blasts or Auer rods, and 0-1 platelets per high-power field. Bone marrow biopsy and aspirate of the right iliac bone demonstrated a hypercellular marrow with erythroid predominant trilineage hematopoiesis with maturation, mild megakaryocytosis, and decreased iron compatible with a diagnosis of ITP (Figure 2). The myeloperoxidase staining was performed to help exclude myeloid neoplasms and rule out ITP mimickers such as acute myeloid leukemia and myelodysplastic syndrome, especially given the history of anemia, bruising, and epistaxis. Flow cytometry of the bone marrow aspirate showed no diagnostic abnormalities, and fluorescence in situ hybridization was normal. Furthermore, FLT3-ITD and FLT3-TKD mutations were both negative. BCR-ABL1 fusion transcripts (P210 and P190) were not detected.

**Table 1:** Laboratory values before treatment initiation Select laboratory value results from hematology and general chemistry blood testing, with reference ranges included ALT, Alanine aminotransferase; AST, Aspartate aminotransferase; LDH, Lactate dehydrogenase; TIBC, Total iron binding capacity; WBC, White blood cell

Laboratory test parameter	Value	Reference range
WBC	20.01	3.8-10.5 K/μL
Neutrophil (%)	82.10	43%-77%
Hemoglobin	2.5	11.5-15.5 g/dL
Hematocrit	10.90	34.5%-45%
Mean cell volume	67.3	80-100 fL
Platelets	7	150-400 K/μL
Uric acid	8.2	2.5-7 mg/dL
LDH	566	50-242 U/L
AST	252	10-40 U/L
ALT	222	10-45 U/L
Iron	24	30-160 µg/dL
TIBC	334	220-430 µg/dL
% saturation, iron	7	14%-150%
Ferritin	22	13-330 ng/mL
Haptoglobin	86	34-200 mg/dL
Reticulocyte percent	11.20	0.5%-2.5%
Absolute reticulocytes	170.9	25-125 K/μL

The patient received six units of packed RBCs and three units of platelets. After transfusion, the hemoglobin increased to 7.9 g/dL; however, the platelet count remained critically low at 2 K/μL, indicating persistent thrombocytopenia. The patient initially received IVIG 1 g/kg for two days and dexamethasone 40 mg for four days to treat suspected ITP, given the lack of improvement with platelet transfusions. She also received iron sucrose for severe iron-deficiency anemia. Despite IVIG and steroids, she remained refractory with a platelet count of 2 K/μL. She then received Promacta 50 mg once daily, but her platelet count did not improve after four days of treatment. Patient was subsequently switched to Nplate 1 mcg/kg weekly. Her platelet count remained at 1 K/μL, so rituximab was started. Of note, the patient was also experiencing a cough for three days and a mild headache that resolved with over-the-counter acetaminophen approximately 1.5 weeks after her initial presentation. A respiratory panel using polymerase chain reaction to detect influenza A, influenza B, SARS-CoV-2, and RSV was performed. The patient tested positive for RSV, which was thought to potentially contribute to her refractory ITP. She developed lower extremity petechiae and mild, bright red blood per rectum, so she was started on Amicar. Her bloody stool likely was due to a lower tract gastrointestinal bleed, given her severely low platelet count, along with the absence of anal fissures or external hemorrhoids on physical examination. Her platelet count remained between 1 K/μL and 6 K/μL, so her Nplate dose was increased to 3 mcg/kg weekly, and prednisone 1 mg/kg daily was added. Her platelet count remained at 3 K/μL, so she was started on cyclosporine 150 mg twice daily. After four days of cyclosporine, her platelet count steadily increased to 22 K/μL, and she was discharged. She was then continued on 150 mg of cyclosporine twice a day after discharge. A repeat platelet count approximately one month after discharge was 30.6 K/μL.

## Discussion

This case highlights a rare and clinically significant instance of severe, refractory ITP in an otherwise healthy adult, likely triggered or exacerbated by acute RSV infection. Although RSV has previously been associated with thrombocytopenia in pediatric patients, the role of ITP as a potential mechanism has not been described in either the pediatric or adult population [6]. Thus, this report expands the clinical spectrum of RSV infection in being a potential immune trigger for severe autoimmune thrombocytopenias, even in immunocompetent adults.

Thrombocytopenia in adults is commonly implicated in hematologic malignancies. However, a previous case described a 28-year-old male patient with a history of acute lymphoblastic leukemia (ALL)-induced thrombocytopenia successfully treated with a double umbilical cord blood transplant. Subsequent cytogenetic analysis revealed a normal karyotype and resolution of thrombocytopenia. However, he presented 19 months later with a new onset of severe thrombocytopenia in the setting of confirmed active RSV infection. Prompt bone marrow evaluation was performed, given the history of ALL, and a new cytogenetic abnormality was detected, involving a translocation between chromosomes 1 and 14. Seven months later, repeat cytogenetic analysis and hematologic workup revealed no apparent chromosomal abnormalities and resolution of thrombocytopenia [7]. Thus, this rare case may suggest a potential etiology of RSV-related thrombocytopenia, although the exact relationship between RSV and autoimmune thrombocytopenia remains unclear.

The patient presented with profound thrombocytopenia, microcytic anemia, and mucocutaneous bleeding. Proposed mechanisms of virus-induced ITP include molecular mimicry, immune complex deposition, and impaired immune tolerance [8]. Despite early administration of standard therapies such as IVIG, corticosteroids, rituximab, and TPO-RAs, this patient remained refractory. Only after initiating cyclosporine, an immunosuppressant not routinely used in early ITP management, did platelet counts begin to improve from three to 22 over the course of four days. While most ITP cases respond to these standard therapies, this patient’s eventual improvement with cyclosporine suggests an alternative pathway of immune dysregulation, potentially amplified by RSV-mediated activation of autoreactive lymphocytes [8]. Refractoriness to multiple lines of therapy further emphasizes the complexity of this immune response.

Of note, our patient also demonstrated acute, severe microcytic anemia, with a hemoglobin count of 2.5 g/dL, a hematocrit of 10.9%, and a mean cell volume of 67.3 fL. Her anemic presentation was improved drastically with six units of packed RBCs. The most common etiology of microcytic anemia is iron deficiency with either blood loss (e.g., gastrointestinal bleeds), dietary lack, or defective iron-absorptive capacities [9]. Other potential causes include inherited thalassemias and anemia of chronic illness. There is currently an unclear relationship between RSV and microcytic anemias. Although certain viral infections may trigger aplastic anemia, as with the case of human parvovirus B19, the relationship between RSV and microcytic anemia remains to be further elucidated [10]. As mentioned previously, a proposed mechanism for thrombocytopenia in the setting of viral illness is molecular mimicry, in which viral antigens may share common epitopes with platelet antigens, leading to their destruction via cross-reacting antibody generation [8]. However, this study did not specifically research RSV. Another study suggested that platelets are specifically used in the clearance of RSV infection via reduced monocyte infection and activation [11]. They also found RSV viral particles within platelets, suggesting the internalization of RSV by platelets. They concluded that thrombocytopenia can predispose patients to worsened RSV infections; however, they did not note resultant thrombocytopenia as a result of RSV infection. Thus, although our case report is consistent with the limited available literature demonstrating a correlation between thrombocytopenia and RSV, further research is needed to infer causality and elucidate the mechanism behind thrombocytopenia.

This single case limits generalizability, and further investigation is needed to elucidate RSV’s role in ITP pathogenesis. Larger systematic studies on virus-associated ITP may help identify risk factors and inform more targeted treatment approaches, including the consideration of alternative agents such as cyclosporine in refractory cases. Clinicians should consider viral triggers, including RSV, in cases of new onset or worsening acute ITP, particularly those unresponsive to standard therapies. In such refractory cases, cyclosporine may serve as a viable salvage therapy.

## Conclusions

This case report represents an unusual, severe exacerbation of ITP potentially due to an underlying RSV infection. RSV infections are typically mild in healthy adults with no concurrent comorbidities such as diabetes, chronic obstructive pulmonary disease, or an immunocompromised state. Severe RSV complications seldom occur, and there are few reports of severe ITP exacerbations associated with underlying RSV infections. ITP may be asymptomatic in nature but can present with skin and mucosal petechiae and bruising, occult gastrointestinal bleeds, and, in severe cases, may lead to intracranial hemorrhage. Thus, severe ITP, especially cases that are refractory to first-line treatments such as corticosteroids and IVIG, must be carefully managed to achieve optimal patient outcomes. Furthermore, a better understanding of the relationship between RSV infection and ITP is critical, as RSV is a common infection globally.
